# A Demi-Decade of Mammal Research: A Rapid Assessment within the Heart of Borneo in Sabah

**DOI:** 10.21315/tlsr2023.34.1.14

**Published:** 2023-03-31

**Authors:** Mohd. Aminur Faiz Suis, Jabanus Miun, Lawrence Tingkoi, Alexander Yuk Loong Hastie, Arthur Chung Yaw Chyang, Reuben Nilus

**Affiliations:** Forest Research Centre, Sabah Forestry Department, P.O. Box 1407, 90715 Sandakan, Sabah, Malaysia

**Keywords:** Heart of Borneo, Illegal Hunting, Rapid Survey, Terrestrial Mammals, Heart of Borneo, Pemburuan Haram, Tinjauan Pantas, Mamalia Daratan

## Abstract

Sabah contributes 4.2 million hectares to the total Heart of Borneo (HoB) areas. Some of the forest reserves in the HoB are newly gazetted as Totally Protected Forest. Hence, their mammal diversity has to be comprehensively documented. This study aims to record the presence of terrestrial mammal species, and assess the prevalence of poaching in selected forest reserves within the Sabah HoB area. A total of 15 forest reserves were surveyed within a 5-year timeframe which recorded 60 terrestrial mammal species, including 21 Bornean endemics. The variation in total enumerated mammal species in the study sites may be derived from unequal sampling efforts, geographical factors and anthropogenic influences. The intensity of poaching within the study sites is high. Though this study is a rapid assessment, it created baseline information for mammal diversity in some of the least studied forest reserves in Sabah, important for conservation of its terrestrial mammals.

## Abstract (fr)

Sabah menyumbang 4.2 juta hektar kepada keluasan kawasan Heart of Borneo (HoB). Beberapa hutan simpan dalam kawasan HoB baru sahaja diwartakan sebagai Kawasan Terlindung Sepenuhnya. Justeru, kepelbagaian mamalia dalam kawasan hutan simpan tersebut perlu didokumentasi secara menyeluruh. Kajian ini bertujuan merekod kewujudan mamalia daratan dan menilai kelaziman aktiviti pemburuan haram di hutan simpan terpilih dalam kawasan HoB di Sabah. Sejumlah 15 hutan simpan telah ditinjau dalam tempoh masa lima tahun. Sebanyak 60 spesies mamalia daratan termasuk 21 spesies endemik di Borneo telah direkodkan. Perbezaaan jumlah spesies mamalia yang direkod di hutan simpan terpilih mungkin disebabkan oleh ketidaksamaan usaha pensampelan, faktor geografi dan tekanan antropogenik. Intensiti pemburuan haram dalam kawasan kajian adalah tinggi. Walaupun kajian ini hanyalah tinjauan pantas, namun ia menghasilkan maklumat asas kepelbagaian mamalia dalam hutan simpan yang kurang diterokai serta menunjukkan kebergunaan pendekatan tinjauan pantas dalam memulihara mamalia daratan.

**Highlights**
- A total of 60 mammal species including 21 Bornean endemics were recorded in 15 forest reserves within the Sabah Heart of Borneo area.
- This mammal rapid survey provides some prerequisite information prior to the implementation of a conservation plan of the understudied reserves.
- The need to increase surveillance and enforcement of forest reserves as part of conservation effort.

## INTRODUCTION

Protection of Bornean faunal diversity has been a major concern among conservationists (Payne & Davies 2013). Illegal hunting of animals (henceforth referred to as poaching) and deforestation are regarded as main threats to wildlife on the island (Phillipps & Phillipps 2018). The death of Malaysia’s last Sumatran rhino delivers a strong signal that the conservation struggle is far from over, and that it is not an easy task - even with active efforts by Government agencies, foreign expertise or help from non-governmental organizations (NGOs). Fortunately, the Malaysian Government through the Sabah State Government’s Forestry Department is still willing to invest much of its resources by implementing various strategies to conserve wildlife diversity in the state (SFD 2013).

The Heart of Borneo (HoB) is a conservation initiative encompassing approximately 22 million hectares (ha) of inter-connected rainforest, across three countries on the island of Borneo; Malaysia (Sabah and Sarawak), Indonesia (Kalimantan) and Brunei Darussalam. This initiative is a joint agreement by governments of respective countries to conserve the biodiversity of the said areas, through sustainable management of forests and other sustainable land uses (WWF 2020). The Strategic Plan for Sabah’s HoB Initiative (2014–2020) involves prioritising collective scientific studies, and enhancing wildlife protection (SFD 2013).

Sabah’s HoB area is approximately 4.2 million ha, comprising at least 95 forest reserves. Overall, there are 333 forest reserves in the state. A number of these forest reserves or conservation areas have received substantial attention from wildlife biologists including Deramakot Forest Reserve (FR) (Samejima *et al.* 2012), Ulu Segama FR (Bernard *et al.* 2016), Danum Valley Conservation Area (Hanya *et al.* 2020) and Kinabalu National Park (Camacho-Sanchez *et al.* 2019). However, some forests are understudied, especially those that are newly classified as Protected Forests and small as well as isolated forest reserves. Prior to 2016, mammal diversity in some of these reserves has never even been scientifically documented. This highlights the urgency for a collective survey of these sites.

Since Sabah’s HoB area comprises many forest reserves, reliable and cost-effective approaches for swiftly surveying mammal diversity are required. Rapid biodiversity assessment is one of the common solutions to meet a wide range of conservation needs (Larsen 2016). In Malaysia alone, mammal surveys have been carried out based on rapid assessment with a sampling period ranging between 7 to 12 days (Zahidin *et al.* 2016; William-Dee *et al.* 2019).

This study is part of the HoB Scientific Expedition series that was organised by the Forest Research Centre of Sabah Forestry Department from 2016 to 2020. Our main objective was to rapidly survey the presence of terrestrial mammal (including arboreal species) in selected forest reserves within the Sabah HoB area. We excluded Chiropteran in this study. Indirectly through this study, we were also able to assess the prevalence of poaching activities in each reserve. This study was designed to create baseline information aside from updating the current distribution data of terrestrial mammal species in Sabah, facilitating an effective collaboration between scientists and policy makers in formulating a comprehensive, data-driven forest management plan.

## MATERIALS AND METHODS

### Study Sites

**Figure 1:**
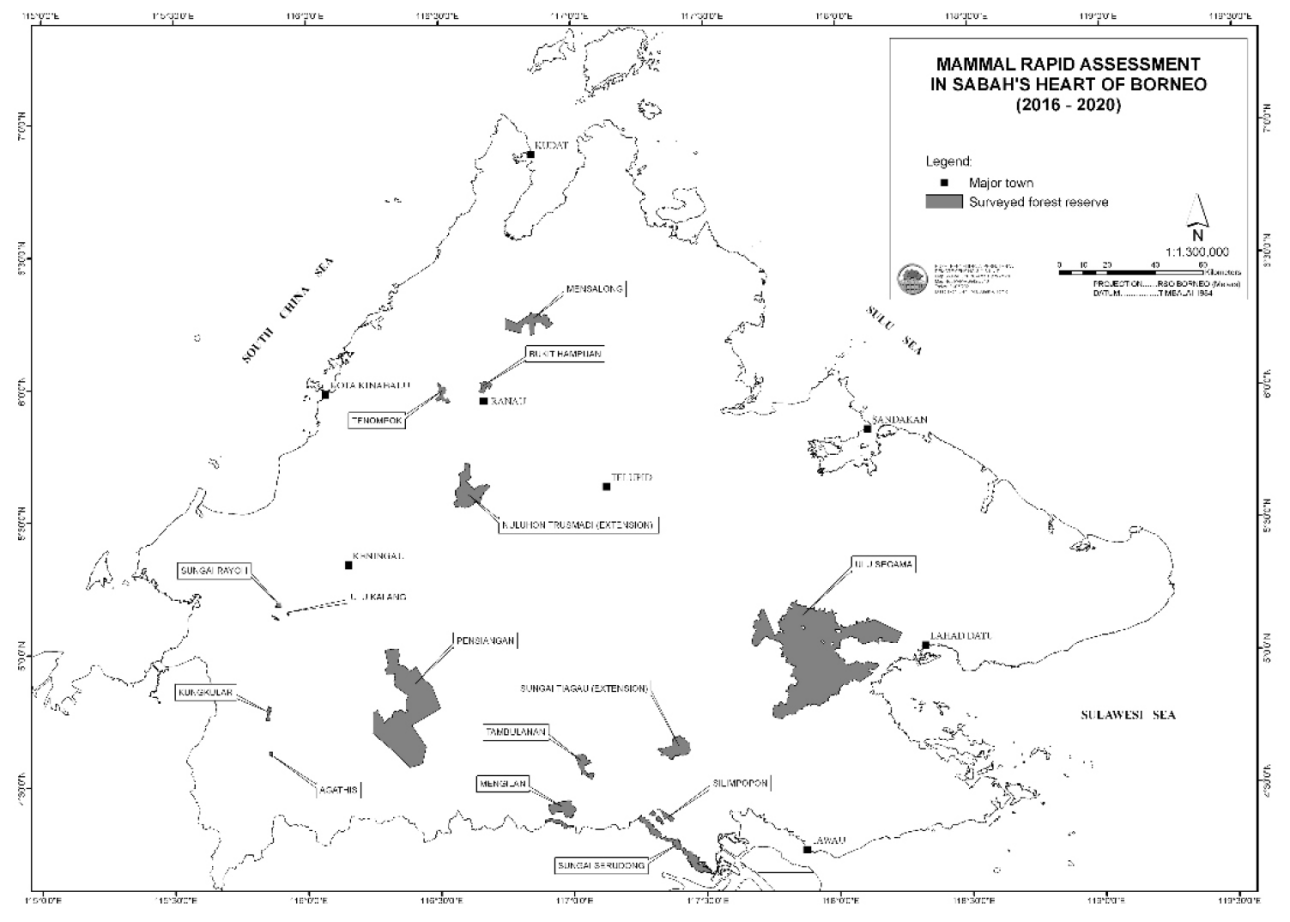
Location of surveyed forest reserves throughout the mammal rapid assessment between 2016 until 2020.

We surveyed 15 forest reserves between 2016 until 2020 (Fig. 1). All forest reserves classified as Class I Protection Forest except the Agathis FR (Class VI Virgin Jungle Reserve). Both classes are meant for environmental protection, hence no timber extraction allowed. Aside from environmental protection, Virgin Jungle Reserve is also conserved for forestry research purposes. This study focused only on specific sites in each reserve. For example, the study site in Ulu Segama FR was on the upstream of Sungai Juak, whereas in Pensiangan FR, the fieldwork was conducted at Sungai Karilin and its surrounding areas.

Forest ecosystem types in each study site varied between mixed dipterocarp, montane, heath and ultramafic forests. Generally, all 15 forest reserves have been disturbed in the past but patches of old-growth forest are still remnant in the study sites. All sites are accessible, though sometimes different modes of transportation (e.g. boat) were utilised.

### Data Collection of Terrestrial Mammal

We recorded the presence of terrestrial mammals using three methods, namely day transect, night spotlight and camera trapping surveys. We conducted day transect survey in all study sites but time spent and distance surveyed varied (Table 1). Both day transect and night spotlight surveys were carried out either along the old-logging roads, asphalt roads, old trails, newly-made trails and rivers. We included the use of a binocular (NIKON Monarch 7) and a white light spotlight in day survey and night survey, respectively. We used a Toyota 4WD Single Cab throughout the night spotlight surveys. We also used a boat to survey mammals along the Serudong river in Sungai Serudong FR. The day survey ranged from 07:30 to 17:30 hours, meanwhile the night spotlight surveys are often executed from 19:30 to 22:00 hours. Both direct (e.g., opportunistic sighting) or indirect (e.g. footprints, presence of dung or nests) sightings were identified by J. Miun and L. Tingkoi, whom have been continuously involved in Bornean wildlife research since 1996.

The inclusion of using camera traps was intended to maximise mammal detection rates throughout this study. We deployed a total of 60 camera traps (Bushnell Trophy Cam HD Aggressor No Glow) in the selected study sites (Table 2). No camera trapping surveys were conducted in Kungkular FR, Agathis FR, Pensiangan FR, Tenompok FR, Sungai Rayoh FR and Silimpopon FR because of inevitable constraints (i.e. equipment availability, time and manpower). One of the camera traps deployed in Sungai Tiagau (Extension) FR went missing; we suspect theft, while another unit in Mengilan FR malfunctioned.

The distribution of camera traps in the study sites was purposively planned in relation to accessibility, safety and time. The location of two camera traps were kept at a minimum distance of 100 m apart. We standardised the position of the camera trap; secured at the base of a tree; 0.5 m above the ground. These passive infrared-activated camera traps are motion-sensitive in that they are triggered to take three shots every time movement is detected. Time interval between two images was set at 30 sec apart. We did not use baits, and set the camera traps to operate for 24 h on a daily basis. We calculated the camera trap success rate in each study site based on Ancrenaz *et al.* (2012).

We followed the IUCN Red List (IUCN 2021) to determine the taxonomic names of each recorded mammal. We classified the conservation status of recorded species based on the IUCN Red List (IUCN 2021) as well as their local protection status in accordance to Sabah’s Wildlife Conservation Enactment 1997 (Amendment 2017) (WCE 1997). In order to assess the prevalence of poaching, we selected eight elements as indicators of the illicit activity: presence of (1) gunshot sounds, (2) used bullet cartridges, (3) shelter or campfires, (4) snares, (5) hunting dogs, (6) game animal carcass, (7) poachers and (8) sale of bushmeat in the vicinity of the forest reserve. We recorded the presence of these elements throughout the rapid survey. We did not employ any interview technique for this study.

## RESULTS

More than half of the surveyed forest reserves are situated in the interior and Tawau division of Sabah. Agathis, Pensiangan, Nuluhon Trusmadi (Extension), Tambulanan, Sungai Serudong, Silimpopon, Sungai Tiagau (Extension) and Mengilan forest reserves are at least 50 km away from any major towns. Journey into the study sites usually involved extensive driving and trekking. For instance, we had to walk for approximately 2.5 h to reach the Agathis FR in Tenom district. The other listed reserves are in close proximity to major towns.

Within the 62 days of field campaigns, we managed to survey a sum of 117.44 km long transects (Table 1). The longest distance surveyed during both day transect and night spotlight methods were in Mengilan FR, while the shortest transect was established in Silimpopon FR. We attained 1,481 trap-nights from 60 camera traps that were deployed in nine study sites (Table 3). Highest mean of camera trap success rate was recorded in Nuluhon Trusmadi (Extension) FR. Duration of the combined survey methods was unequal across the study sites. The extended sampling duration in Mengilan FR is a result of the first Movement Control Order implemented during the COVID-19 pandemic.

Throughout the rapid assessment, we recorded a total of 60 terrestrial mammal species belonging to nine orders, 23 families and 43 genera including 21 Bornean endemics (Table 4; Appendix A). Rodentia was the most speciose order with 18 species, followed by Carnivora (15 species) and Primates (10 species). The top four most speciose families were Sciuridae (15 species), Cercopithecidae (6 species), Viverridae (6 species) and Tupaiidae (6 species). Of the 60 recorded mammal species, 22 of them are categorised as threatened species (i.e. Critically Endangered, Endangered and Vulnerable) based on their global conservation status (IUCN 2021). More than half of the recorded mammals are listed under Schedule 1 (totally protected species) or Schedule 2 (protected species) of the Sabah’s Wildlife Conservation Enactment 1997 (Amendment 2017).

**Figure 2:**
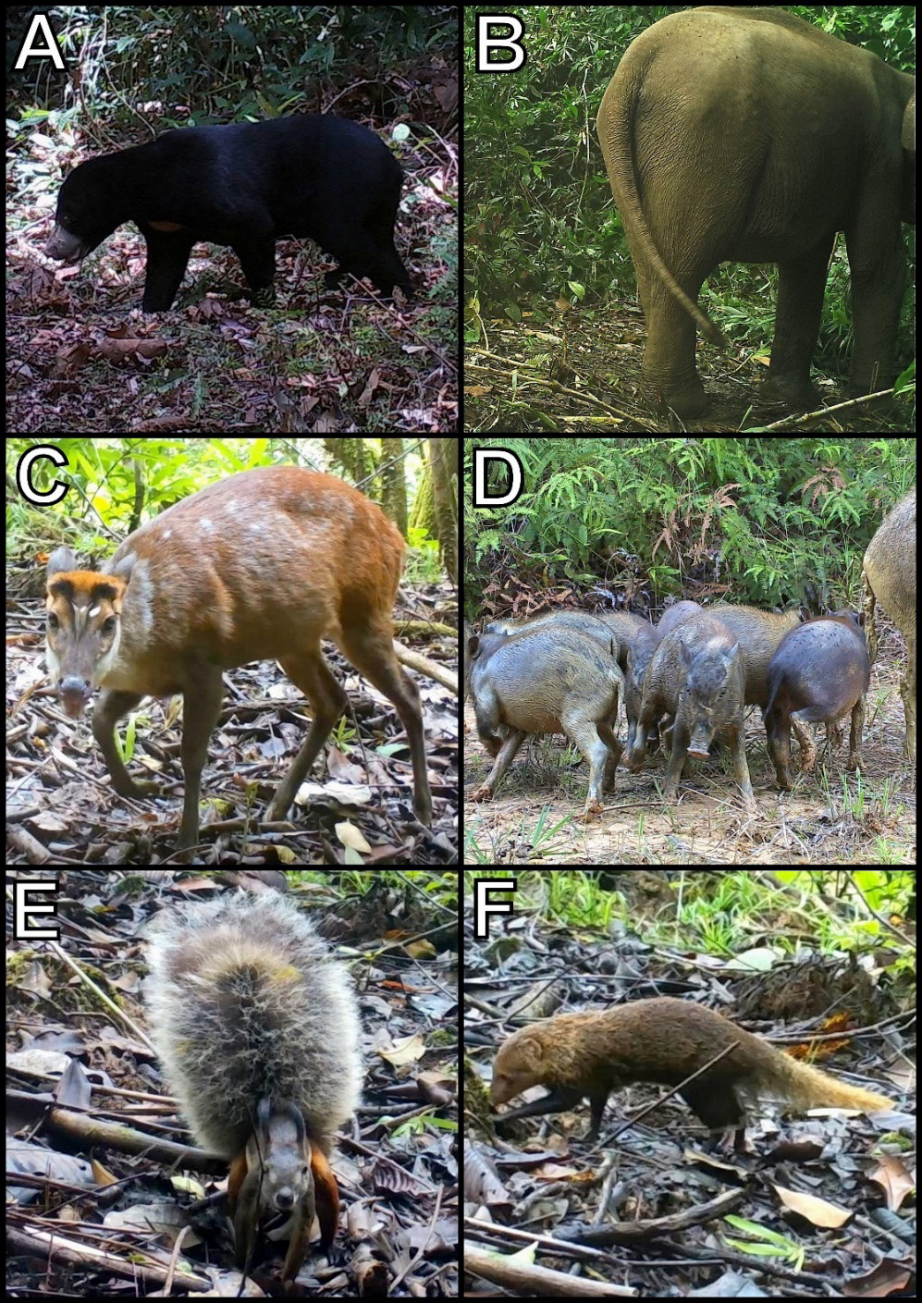
Selected photographic data derived from camera trapping method throughout the mammal rapid assessment between 2017 until 2020: (A) Sun bear (*Helarctos malayanus*), (B) Asian elephant (*Elephas maximus*), (C) Red muntjac (*Muntiacus muntjak*), (D) Bearded pigs (*Sus barbatus*), (E) Tufted ground squirrel (*Rheithrosciurus macrotis*) and (F) Short-tailed mongoose (*Herpestes brachyurus*).

A total of 43 species were detected through camera trapping (Fig. 2). The day transect survey as well as the night spotlight survey recorded 46 species and 19 species, respectively. There were ten species recorded by all three methods, namely the bearded pig, Asian elephant, leopard cat, common palm civet, greater mousedeer, lesser mousedeer, Malay civet, red muntjac, sambar deer and Western tarsier.

We enumerated 36 mammal species in Mengilan FR which is the highest among all study sites (Table 4). Fieldwork in Silimpopon FR resulted in the discovery of six mammal species. On average, rapid assessment in each reserve recorded the presence of 13 species. In most reserves, we registered 14 to 23 species.

**Figure 3:**
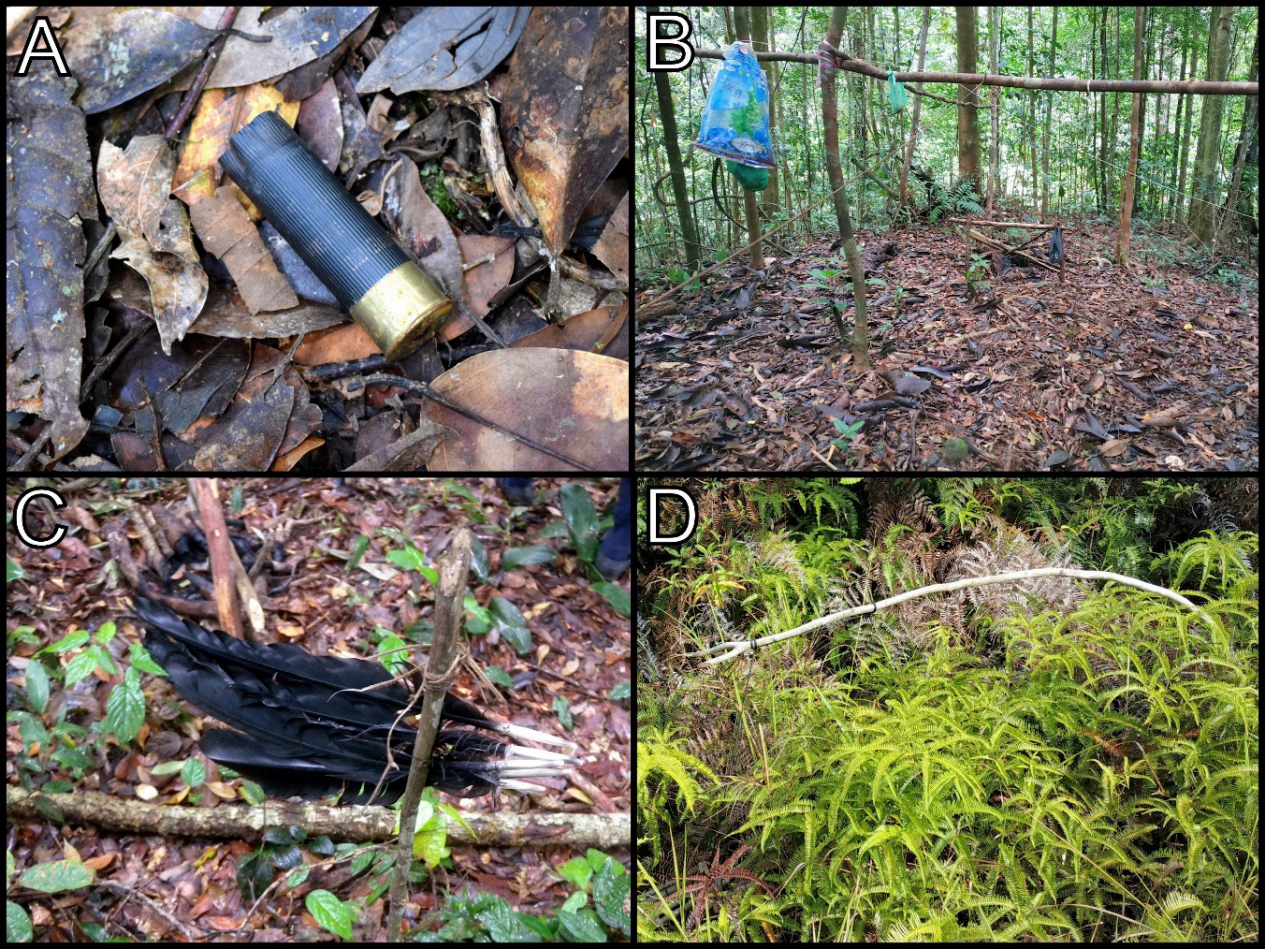
Examples of the poaching elements observed throughout the mammal rapid assessment between 2016 until 2020: (A) Used bullet cartridge in Pensiangan Forest Reserve; (B) Abandoned shelter in Bukit Hampuan Forest Reserve; (C) Bird carcass and campfire in Kungkular Forest Reserve and (D) Active snare in Sungai Serudong Forest Reserve.

None of the surveyed forest reserves were devoid of poaching elements (Table 5; Fig. 3). All eight elements were present in Sungai Tiagau (Extension) and Pensiangan forest reserves. The presence of poachers was confirmed based on their footprints and vehicle tyre tracks. Occasionally, we even spotted the poachers during field campaigns. In Pensiangan FR, poachers were sighted on motorcycles without a license plate number while carrying dead bearded pigs. Other encounters occurred in Bukit Hampuan, Sungai Serudong, Kungkular, Nuluhon Trusmadi (Extension), Mengilan and Tambulanan forest reserves. Another common poaching element was the presence of snares. Most of these snares are designed to catch cervids, pigs, porcupines and pangolins. We also found empty bullet cartridges, abandoned shelters and game animal carcasses in most of the study sites.

## DISCUSSION

Borneo is a home to 147 terrestrial mammals, not including the order Chiroptera (Phillipps & Phillipps 2018). Therefore, this study has recorded about 40% of the total terrestrial species found on the island. The high number of unrecorded species is because of the nature of our sampling design – rapid assessment. Reporting species presence, many for the first time at certain sites, is important to verify current and historic distribution of mammals in Sabah (Kramer-Schadt *et al.* 2016).

All forest reserves were under-sampled and hence, the list of recorded species from each reserve is not comprehensive. Indeed, more species are to be recorded if sampling efforts were increased. For instance, Ulu Segama and its surrounding areas are known to harbour at least 58 terrestrial mammal species (SFD 2016) as opposed to only 18 species during the rapid survey in the upstream area of Bole River in the same forest reserve.

In Mengilan FR, the highest number of recorded species might have been a result of the longer sampling period. However, we do not exclude other factors including location and impact of human activities. Severe anthropogenic disturbance in areas close to major towns has reduced mammal diversity in Peninsular Malaysia (William-Dee *et al.* 2019). Even though the duration of survey in Sungai Tiagau (Extension), Sungai Serudong and Tambulanan forest reserves were among the lowest, total recorded mammals from these reserves still ranged between 20 to 23 species. These aforementioned forest reserves are further from Tawau town but are closer to the Malaysia-Indonesia border. Fieldwork in Ulu Kalang FR which is just 3 km away from Tenom town yielded only 10 species.

Forest connectivity also led to the variation in total enumerated mammals across study sites. Fieldwork in Silimpopon FR focused only on its second block (477.37 ha). This block is completely isolated and is surrounded by oil palm plantation which may have contributed to its low species richness. Our finding corroborates with a study conducted in Lahad Datu where fragmented forests recorded low mammal species richness (Bernard *et al.* 2014). The majority of the study sites are connected to other forest reserves of different classes. Ulu Segama FR and Mengilan FR formed contiguous forest covers with Danum Valley Conservation Area as well as the vast North Kalimantan rainforest, respectively.

Other factors that influenced the detection of mammal species were potentially altitude and forest quality. It is reported that small mammal diversity decreases from low altitudes on both Mount Tambuyukon and Mount Kinabalu (Camacho-Sanchez *et al.* 2019). Similarly, our fieldwork in the montane environment (1040 m–1650 m) of Tenompok FR recorded 10 mammal species. At a much lower altitude, we detected seven species from Sungai Rayoh FR. It can be explained due to the recurrence of fire incidents in this reserve which formed the current extensive secondary vegetation subsequently impacting mammal richness. Detrimental impacts of forest fire on mammal population have also been reported in other parts of Borneo (Rijksen & Meijaard 1999).

Since the sampling efforts through camera trapping, day transects and night spotlight surveys were different across study sites, we could not confidently determine their sampling efficacies. However, wildlife ecologists favour camera trapping due to its effectiveness (Wearn & Glover-Kapfer 2019). We frequently detected more mammal species via day transect and night spotlight surveys. In some cases, our camera traps either malfunctioned or were stolen. Despite that, the combination of these three methods ultimately increased the mammal detection rates of this study.

Undeniably, mammal communities in Sabah are pressured by illegal hunting. Signs of poaching activities have become the norm throughout our field campaigns from 2016 to 2020. Illegal hunting of wildlife has also been reported outside the surveyed forest reserves (SFD 2016). In Sabah, poaching has been associated with indigenous culture (Wong *et al.* 2012), traditional medicine (Gomez *et al.* 2020), trade (Pantel & Anak 2010) and bushmeat (Kurz *et al.* 2020). Our encounters with poachers have so far never resulted in any undesirable situation. Many of the encounters happened while we were in a vehicle, travelling back and forth to the study sites. We however, tried to discourage some of them from proceeding with their illicit activities.

We reported the presence of poaching elements in just 15 out of 333 forest reserves. This leaves us to ponder – do we have enough manpower to deter poaching in Sabah? Sabah Forestry Department has implemented a number of initiatives to tackle the poaching issue. In 2016, SFD Protect Team was launched to strengthen the department’s capability in combating forest crimes. In order to increase manpower capacity, local communities were encouraged to help through the Honorary Forest Rangers programme. Fortunately, various NGOs are also partnering with the Government to be actively involved in mitigating illegal harvest of forest resources in Sabah. For instance, Sabah Environmental Trust has been closely involved in patrolling Danum Valley, Maliau Basin and Imbak Canyon conservation areas since 2017 (SFD 2019). These efforts are bearing fruit because 860 cases of forest crimes were reported between 2016 to 2020 which resulted in the arrest of 433 individuals and 154 of them were convicted (SFD 2020).

In addition to that, the Sabah Forestry Department has continuously worked on instilling public conservation awareness. A series of environmental education programmes have been carried out by the department through its Rainforest Discovery Centre since the implementation of the HoB initiative. Such programmes create opportunities for local communities to support government efforts in conservation (Arpa *et al*. 2016). Because of the existence of local settlements nearby the study sites, we highly recommend recruiting the participation of these communities in the form of citizen science. In Sabah, citizen scientists have positively contributed to conservation efforts when scientific knowledge was expanded to them (Freitag *et al.* 2018; Araujo *et al.* 2020). Lastly, conservation programmes should also prioritise the improvement of forest connectivity of fragmented reserves. If left unchecked, it could possibly lead to the loss as a functionally connected habitat (Ocampo-Peñuela *et al.* 2020).

## CONCLUSION

This study documented the presence of 60 species across 15 study sites, which indirectly updated the distribution of terrestrial mammal species in Sabah. We concluded that the actual number of species in each forest reserve is actually way higher, and hence, this study should not be used to confirm the absence of any terrestrial mammal species. This study also reiterated the rampant poaching issues in this state. In future, fieldwork should be carried out at a larger scale with focus on the understudied sites. In the long run, mammal rapid assessment could trigger a more concrete and targeted conservation program that emphasise protection of threatened species and ecosystems.

## Figures and Tables

**Table 1:** Summary of transect and night spotlight surveys throughout the mammal rapid assessment between 2016 until 2020.

Forest reserve	Range of survey	Survey days	Duration of fieldwork	Distance (km)	Remark
Kungkular	March 2016	3	8h	2	Day trek
Agathis	March 2016	1	2h	1.1	Day trek
Pensiangan	July 2016	4	5h 4m	2.7	Day trek
Tenompok	September 2016	4	7h	2.5	Day trek
Ulu Segama	April 2017	3	5h 44m	3.7	Day trek
Nuluhon Trusmadi (Extension)	May 2017	5	18h 8m	8.4	Day trek
		1	2h 37m	10	Night survey
Tambulanan	October 2017	3	9h 10m	3.4	Day trek
		1	1h 10m	5.2	Night survey
Ulu Kalang	February 2018	2	10h 42m	4.5	Day trek
Sungai Rayoh	February 2018	1	3h 22m	1	Day trek
Mensalong	July–August 2019	4	14h 55m	4.3	Day trek
Sungai Serudong	September–October 2019	8	15h 40m	7.03	Day trek
		1	1h 20m	16.6	By boat
Silimpopon	September 2019	1	3h	0.8	Day trek
Sungai Tiagau (Extension)	November 2019	5	11h 46m	4.73	Day trek
Mengilan	February–August 2020	7	24h 7m	8.36	Day trek
		2	3h 45m	26.6	Night survey
Bukit Hampuan	September 2020	6	14h 44m	4.52	Day trek

**Table 2:** Summary of camera trapping survey throughout the mammal rapid assessment between 2017 until 2020.

Forest reserve	Total camera traps	Range of survey	Altitude (masl)
Ulu Segama	5	March–April 2017	202–241
Nuluhon Trusmadi (Extension)	5	May 2017	825–1039
Tambulanan	5	September–October 2017	376–509
Ulu Kalang	3	February–March 2018	715–790
Mensalong	10	July–August 2019	588–943
Sungai Serudong	9	September–October 2019	14–145
Sungai Tiagau (Extension)	10	November 2019	225–607
Mengilan	9	February–June 2020	591–1160
Bukit Hampuan	4	August–September 2020	910–1450

**Table 3:** Result of camera trapping survey throughout the mammal rapid assessment between 2017 until 2020.

Forest reserve	Total trap-nights	Total captured images	Total independent mammal images	Mean of camera trap success rate (per 100 trap nights)
Ulu Segama	42	879	28	67.22
Nuluhon Trusmadi (Extension)	45	431	62	137.78
Tambulanan	35	314	40	114.28
Ulu Kalang	48	274	55	114.60
Mensalong	173	4359	106	70.84
Sungai Serudong	32	374	50	129.44
Sungai Tiagau (Extension)	48	494	61	131.33
Mengilan	1008	8280	1073	106.87
Bukit Hampuan	50	360	47	88.45

**Table 4:** Total enumerated species in each forest reserve throughout the mammal rapid assessment between 2016 until 2020.

Forest reserve	Total recorded species
Kungkular	11
Agathis	14
Pensiangan	16
Tenompok	10
Ulu Segama	18
Nuluhon Trusmadi (Extension)	17
Tambulanan	23
Ulu Kalang	10
Sungai Rayoh	7
Mensalong	20
Sungai Serudong	20
Silimpopon	6
Sungai Tiagau (Extension)	21
Mengilan	36
Bukit Hampuan	16

**Table 5:** Elements of poaching activity observed in each forest reserve throughout the mammal rapid assessment between 2016 until 2020.

Element	Forest Reserve														

	A	B	C	D	E	F	G	H	I	J	K	L	M	N	O
Gunshot sound		✓	✓			✓			✓	✓	✓	✓			
Used bullet cartridge	✓	✓	✓	✓	✓	✓	✓	✓	✓		✓	✓	✓	✓	✓
Shelter or campfire	✓	✓	✓	✓	✓	✓	✓	✓	✓		✓	✓	✓	✓	✓
Snare	✓	✓	✓	✓	✓	✓	✓	✓	✓	✓	✓	✓	✓	✓	✓
Hunting dog	✓		✓			✓			✓			✓		✓	✓
Game animal carcass	✓	✓	✓	✓	✓	✓	✓	✓	✓		✓	✓	✓	✓	✓
Poacher	✓	✓	✓	✓	✓	✓	✓	✓	✓	✓	✓	✓	✓	✓	✓
Sale of bushmeat in the vicinity of forest reserve			✓				✓		✓				✓	✓	✓

*Notes*: (A) Bukit Hampuan, (B) Mengilan, (C) Sungai Tiagau (Extension), (D) Silimpopon, (E) Sungai Serudong, (F) Mensalong, (G) Kungkular, (H) Agathis, (I) Pensiangan, (J) Tenompok, (K) Ulu Segama, (L) Nuluhon Trusmadi (Extension), (M) Tambulanan, (N) Ulu Kalang and (O) Sungai Rayoh.

## APPENDIX

### Appendix A

List of terrestrial species recorded throughout the mammal rapid assessment between 2016 until 2020.

**Table:**

Scientific name	Common name	WCE 1997	IUCN 2020	Forest Reserve														

				A	B	C	D	E	F	G	H	I	J	K	L	M	N	O
*Elephas maximus*	Asian elephant	S1	EN		✓	✓								✓		✓		
*Aonyx cinerea*	Asian small-clawed otter	S2	VU		✓							✓				✓		
*Hemigalus derbyanus*	Banded civet	S2	NT		✓	✓			✓	✓								
*Prionodon linsang*	Banded linsang	S2	LC						✓					✓	✓		✓	
*Sus barbatus*	Bearded pig	S3	VU	✓	✓	✓	✓	✓	✓	✓	✓	✓		✓	✓	✓	✓	✓
*Arctictis binturong*	Binturong	S2	VU		✓	✓												
*Bos javanicus*	Banteng	S1	EN			✓								✓		✓		
*Callosciurus orestes* *	Bornean black-banded squirrel	NL	LC										✓					
*Melogale everetti* *	Bornean ferret badger	S2	EN										✓				✓	
*Hylobates muelleri* *	Bornean gibbon	S2	EN		✓	✓		✓				✓		✓	✓	✓		
*Dremomys everetti* *	Bornean mountain ground squirrel	NL	LC		✓								✓					
*Pongo pygmaeus* *	Bornean orangutan	S1	CR											✓				
*Muntiacus atherodes* *	Bornean yellow muntjac	S3	NT		✓	✓				✓	✓		✓			✓		
*Paradoxurus hermaphroditus*	Common palm civet	S2	LC	✓	✓	✓		✓	✓	✓						✓		
*Callosciurus adamsi* *	Ear-spot squirrel	NL	NT															
*Tragulus napu*	Greater mousedeer	S3	LC		✓	✓		✓	✓	✓	✓			✓	✓	✓	✓	
*Nycticebus coucang*	Greater slow loris	S2	EN		✓						✓					✓		
*Sundasciurus hippurus*	Horse-tailed squirrel	NL	NT		✓			✓	✓						✓			
*Diplogale hosei* *	Hose’s civet	S2	VU		✓													
*Sundasciurus jentinki* *	Jentink’s squirrel	NL	LC										✓					
*Tupaia tana*	Large treeshrew	NL	LC					✓										
*Exilisciurus exilis* *	Least pygmy squirrel	NL	DD	✓									✓					
*Prionailurus bengalensis*	Leopard cat	S2	LC		✓		✓	✓	✓	✓	✓	✓				✓		
*Tragulus kanchil*	Lesser mousedeer	S3	LC		✓	✓				✓	✓			✓				
*Tupaia minor*	Lesser treeshrew	NL	LC									✓						
*Macaca fascicularis*	Long-tailed macaque	S2	VU	✓	✓			✓						✓	✓			
*Trichys fasciculata*	Long-tailed porcupine	S2	LC	✓	✓				✓							✓	✓	✓
*Sundasciurus lowii*	Low’s squirrel	NL	LC	✓					✓						✓			
*Viverra tangalunga*	Malay civet	S2	LC		✓	✓			✓			✓		✓	✓	✓		
*Hystrix brachyura*	Malayan porcupine	S3	LC		✓	✓		✓	✓	✓	✓	✓				✓		
*Paguma larvata*	Masked palm civet	S2	LC	✓	✓													
*Echinosorex gymnura*	Moonrat	NL	LC					✓								✓		
*Tupaia montana* *	Mountain treeshrew	NL	LC										✓					
*Ratufa affinis*	Pale giant squirrel	S2	NT	✓	✓	✓			✓	✓		✓		✓	✓	✓	✓	✓
*Macaca nemestrina*	Pig-tailed macaque	S2	VU	✓	✓	✓		✓	✓	✓				✓	✓	✓	✓	✓
*Tupaia longipes* *	Plain treeshrew	NL	LC	✓				✓										
*Callosciurus notatus*	Plantain squirrel	NL	LC	✓		✓	✓	✓				✓	✓		✓	✓		
*Callosciurus prevostii*	Prevost’s squirrel	NL	LC			✓		✓	✓			✓						
*Nasalis larvatus* *	Proboscis monkey	S1	EN					✓										
*Petaurista petaurista*	Red giant flying squirrel	S2	LC		✓						✓							
*Presbytis rubicunda* *	Red langur	S2	VU		✓						✓			✓	✓	✓		
*Rusa unicolor*	Sambar deer	S3	VU	✓	✓	✓	✓	✓		✓	✓	✓		✓	✓	✓	✓	✓
*Herpestes brachyurus*	Short-tailed mongoose	S2	NT	✓	✓	✓			✓					✓	✓			
*Trachypithecus cristatus*	Silvered langur	S2	VU					✓			✓							
*Sundasciurus tenuis*	Slender squirrel	NL	LC						✓			✓						
*Tupaia gracilis* *	Slender treeshrew	NL	LC					✓										
*Tupaia dorsalis* *	Striped treeshrew	NL	DD		✓													
*Helarctos malayanus*	Sun bear	S1	VU		✓		✓	✓			✓	✓			✓	✓		
*Neofelis diardi*	Sunda clouded leopard	S1	VU		✓									✓				
*Galeopterus variegatus*	Sunda flying lemur	S2	LC		✓											✓		
*Manis javanica*	Sunda pangolin	S1	CR	✓	✓	✓	✓						✓					
*Mydaus javanensis*	Sunda stink-badger	S2	LC		✓	✓					✓							
*Hystrix crassispinis* *	Thick-spined porcupine	S2	LC		✓				✓								✓	✓
*Aeromys thomasi* *	Thomas’s flying squirrel	S2	LC													✓		
*Rheithrosciurus macrotis* *	Tufted ground squirrel	S2	VU		✓				✓						✓			
*Exilisciurus whiteheadi* *	Tufted pygmy squirrel	NL	LC										✓					
*Cephalopachus bancanus*	Western tarsier	S2	VU						✓			✓						
*Martes flavigula*	Yellow-throated marten	S2	LC	✓	✓	✓								✓				

*Note*: IUCN Red List of Threatened Species (2020) = (CR) critically endangered, (EN) endangered, (VU) vulnerable, (NT) near threatened, (LC) least concern and (DD) data deficient. Sabah Wildlife Conservation Enactment (WCE 1997) (2017 Amendment) = (Schedule 1, S1) totally protected animals, (Schedule 2, S2) protected animals, (Schedule 3, S3) game animals and (NL) not listed. Forest Reserve = (A) Bukit Hampuan, (B) Mengilan, (C) Sungai Tiagau (Extension), (D) Silimpopon, (E) Sungai Serudong, (F) Mensalong, (G) Kungkular, (H) Agathis, (I) Pensiangan, (J) Tenompok, (K) Ulu Segama, (L) Nuluhon Trusmadi (Extension), (M) Tambulanan, (N) Ulu Kalang and (O) Sungai Rayoh.

* Bornean endemic.

## References

[b1-tlsr-34-1-261] Ancrenaz M, Hearn AJ, Ross J, Sollmann R, Wilting A (2012). Handbook for wildlife monitoring using camera-traps.

[b2-tlsr-34-1-261] Araujo G, Ismail AR, McCann C, McCann D, Legaspi CG, Snow S, Labaja J, Manjaji-Matsumoto M, Ponzo A (2020). Getting the most out of citizen science for endangered species such as whale shark. Journal of Fish Biology.

[b3-tlsr-34-1-261] Arpa AN, Petol GH, Joeman BD (2016). Environmental education programme for RAMSAR by Rainforest Discovery Centre, Sabah Forestry Department.

[b4-tlsr-34-1-261] Bernard H, Baking EL, Giordano AJ, Wearn OR, Ahmad AH (2014). Terrestrial mammal species richness and composition in three small forest patches within an oil palm landscape in Sabah, Malaysian Borneo. Mammal Study.

[b5-tlsr-34-1-261] Bernard H, Bili R, Matsuda I, Hanya G, Wearn OR, Wong A, Ahmad AH (2016). Species richness and distribution of primates in disturbed and converted forest landscapes in Northern Borneo. Tropical Conservation Science.

[b6-tlsr-34-1-261] Camacho-Sanchez M, Hawkins MTR, Yu FTY, Maldonado JE, Leonard JA (2019). Endemism and diversity of small mammals along two neighboring Bornean mountains. PeerJ.

[b7-tlsr-34-1-261] Freitag H, Pangantihon CV, Njunjuć I (2018). Three new species of *Grouvellinus* Champion, 1923 from Maliau Basin, Sabah, Borneo, discovered by citizen scientists during the first Taxon Expedition (Insecta, Coleoptera, Elmidae). ZooKeys.

[b8-tlsr-34-1-261] Gomez L, Shepherd CR, Khoo MN (2020). Illegal trade of sun bear parts in the Malaysian states of Sabah and Sarawak. Endangered Species Research.

[b9-tlsr-34-1-261] Hanya G, Kanamori T, Kuze N, Wong ST, Bernard H (2020). Habitat use by a primate community in a lowland dipterocarp forest in Danum Valley, Borneo. American Journal of Primatology.

[b10-tlsr-34-1-261] International Union for Conservation of Nature (IUCN) (2021). The IUCN Red List of Threatened Species.

[b11-tlsr-34-1-261] Kramer-Schadt S, Reinfelder V, Niedballa J, Lindenborn J, Stillfried M, Heckmann I, Wilting A (2016). The Borneo carnivore database and the application of predictive distribution modelling. Raffles Bulletin of Zoology.

[b12-tlsr-34-1-261] Kurz D, Saikim FH, Justine TJ, Bloem J, Libassi M, Luskin MS, Lauren LS, Goossens B, Brashares JS, Potts MD (2020). Oil palm expansion reshapes indigenous hunting: Kadazandusun-Murut bearded pig hunting practices in Sabah, Malaysia (preprint). EcoEvoRxiv.

[b13-tlsr-34-1-261] Larsen TH (2016). Core standardized methods for rapid biological field assessment.

[b14-tlsr-34-1-261] Ocampo-Peñuela N, Garcia-Ulloa J, Kornecki I, Philipson CD, Ghazoul J (2020). Impacts of four decades of forest loss on vertebrate functional habitat on Borneo. Frontiers in Forest and Global Change.

[b15-tlsr-34-1-261] Pantel S, Anak NA (2010). A preliminary assessment of pangolin trade in Sabah.

[b16-tlsr-34-1-261] Payne J, Davies G (2013). Conservation of rain forest mammals in Sabah: long term perspectives. The Raffles Bulletin of Zoology.

[b17-tlsr-34-1-261] Phillipps Q, Phillipps K (2018). Phillipps’ field guide to the mammals of Borneo and their ecology: Sarawak edition.

[b18-tlsr-34-1-261] Rijksen HD, Meijaard E (1999). Our vanishing relative: The status of wild Orang-Utans at the close of the twentieth century.

[b19-tlsr-34-1-261] Sabah Forestry Department (SFD) (2013). Strategic plan of action (Sabah) the Heart of Borneo Initiative 2014–2020.

[b20-tlsr-34-1-261] Sabah Forestry Department (SFD) (2016). Annual report 2016.

[b21-tlsr-34-1-261] Sabah Forestry Department (SFD) (2019). Annual report 2019.

[b22-tlsr-34-1-261] Sabah Forestry Department (SFD) (2020). Annual report 2020.

[b23-tlsr-34-1-261] Samejima H, Ong R, Lagan P, Kitayama K (2012). Camera-trapping rates of mammals and birds in a Bornean tropical rainforest under sustainable forest management. Forest Ecology and Management.

[b24-tlsr-34-1-261] Wearn OR, Glover-Kapfer P (2019). Snap happy: camera traps are an effective sampling tool when compared with alternative methods. Royal Society Open Science.

[b25-tlsr-34-1-261] Wildlife Conservation Enactment (WCE) (1997). Wildlife Conservation Enactment (Amendment 2017).

[b26-tlsr-34-1-261] William-Dee J, Khan FAA, Rosli Q, Morni MA, Azhar I, Lim LS, Tingga RCT, Rahman MRA (2019). Comparative distribution of small mammals diversity in protected and non-protected area of Peninsular Malaysia. Tropical Life Sciences Research.

[b27-tlsr-34-1-261] Wong A, Huaimei Y, Wong C, Shukor JA (2012). A study on hunting activity of sambar deer and bearded pig in Paitan Forest Reserve, Pitas, Sabah, Malaysia. Journal of Tropical Biology and Conservation.

[b28-tlsr-34-1-261] World Wide Fund for Nature (WWF) (2020). Heart of Borneo.

[b29-tlsr-34-1-261] Zahidin MA, Roslan A, Marni W, Kombi M, Abdullah MT, Bako CR (2016). Biodiversity assessment and updated checklist of faunal diversity in Bako National Park, Sarawak, Malaysian Borneo. Journal of Sustainability Science and Management.

